# Association between short-term exposure to ambient air pollution and hospital admissions for transient ischemic attacks in Beijing, China

**DOI:** 10.1007/s11356-020-10963-1

**Published:** 2020-10-03

**Authors:** Runhua Zhang, Yong Jiang, Guitao Zhang, Miaoxin Yu, Yongjun Wang, Gaifen Liu

**Affiliations:** 1 Department of Neurology, Beijing Tiantan Hospital, Capital Medical University, No.119, South 4th Ring West Road, Fengtai District, Beijing, 100070 China; 2 China National Clinical Research Center for Neurological Diseases, Beijing, 10070 China

**Keywords:** Transient ischemic attack, Air pollution, Particulate matter, Time-series, Hospital admissions

## Abstract

Numerous studies have examined the associations between air pollution and stroke. However, little is known about the associations between air pollution and transient ischemic attack (TIA). In this study, we aimed to conduct a time-series study to systematically examine the associations between hospital admissions for TIA and air pollutants. Admissions for TIA (ICD-10: G45) from 1 January 2014 to 31 December 2016 were identified based on the primary diagnosis from 134 hospitals in Beijing, China. Hourly measurements of air pollutants were obtained from the National Air Pollution Monitoring System. Generalized additive models with quasi-Poisson regression were used to determine the associations for each pollutant. Additionally, stratified analysis was implemented to examine whether age, gender, temperature, and season were the potential effect modifiers. Restricted cubic spline was applied to investigate the exposure-response curve. In total, 109,975 hospital admissions for TIA were included. The positive associations were detected between PM_2.5_, PM_10_, SO_2_, NO_2_, and CO and hospital admissions for TIA. The effects of PM_2.5_ and PM_10_ in men are stronger than in women. Additionally, the effects of PM_2.5_, PM_10_, SO_2_, and O_3_ are more pronounced on warm days than cool days. From exposure-response curves, we observe a nearly linear relationship for PM_2.5_, PM_10_, CO, and NO_2_. Further studies are needed to verify the association. This research contributes evidence on the association between air pollution and admissions for TIA in the low- and middle-income countries and may promote related public health policy development.

## Introduction

Air pollution is one of the leading causes of risk-attributable death, accounting for over 4 million worldwide deaths each year (Stanaway et al. 2018). As a potentially modifiable risk factor, the effects of air pollution on human health are a topic that has received much attention in recent decades. A large body of epidemiological and clinical studies supports the existence of positive associations between air pollution and the morbidity and mortality of ischemic stroke, as well as hemorrhagic stroke (Andersen et al. 2012; Huang et al. 2019; Sun et al. 2019; Zhang et al. 2018). According to the International Classification of Diseases (ICD), both transient ischemic attack (TIA) and stroke are categorized as cerebrovascular disorders (Bo Norrving et al. 2013; Sacco et al. 2013). Although clinical and pathological similarities exist between TIA and ischemic stroke, the criteria for clinical diagnosis are distinctive for each, and they are classified as different types of cerebrovascular disorders. Compared with stroke, there is little research on the effects of air pollution on TIA.

TIA is a focal neurologic dysfunction caused by focal brain or retinal ischemia. Typically, there is no evidence of infarction on imaging, and symptoms generally resolve within 24 h (Bo Norrving et al. 2013). TIA has been recognized as a strong predictor of subsequent stroke and is associated with poorer long-term survival (Clissold et al. 2020; Kleindorfer et al. 2005). The risk of subsequent stroke after experiencing a TIA was 17.3% at 3 months (Coull et al. 2004), and the mortality after experiencing a TIA was 12% in 1 year (Kleindorfer et al. 2005). Whether air pollution is the modifiable risk factor for TIA is uncertain. Moreover, the majority of previous studies (Bedada et al. 2012; Lisabeth et al. 2008) were conducted in high-income countries, with limited studies from low- and middle-income countries, where levels and components of various air pollutants were markedly different from high-income countries (Li et al. 2015).

Increased knowledge of risks associated with air pollution would allow for improved implementation of public health intervention strategies. In this study, we aimed to conduct a time-series analysis to systematically assess the association between short-term exposure to air pollution and hospital admissions for TIA. The air pollutants being explored include particulate matter with aerodynamic diameter < 2.5 microns (PM_2.5_), with aerodynamic diameter < 10 microns (PM_10_), carbon monoxide (CO), nitrogen dioxide (NO_2_), sulfur dioxide (SO_2_), and ozone (O_3_).

## Materials and methods

### Data collection

We obtained data on daily hospital admissions from the Hospital Discharge Abstract Database, which is maintained by the Beijing Municipal Health Commission Information Center (HCIC). All hospitals were grade 2 or 3 and had the capability of diagnosing and treating patients with TIA. To improve the accuracy of disease diagnoses, the HCIC applies strict quality control procedures and organized validation programs annually. Using the International Classification of Diseases 10th Revision (ICD-10), all admissions for TIA (G45) were identified based on the primary diagnosis. The time period was 1 January 2014 to 31 December 2016. The daily counts of TIA admissions were also sorted by sex and age.

In addition, we obtained daily concentrations of air pollutants from the National Air Pollution Monitoring System, including PM_2.5_, PM_10_, CO, SO_2_, NO_2_, and O_3_. Hourly measurements of air pollutants were collected from all monitoring stations. Similar to the methods employed by several previous studies, we used average measurements as a proxy for exposure (Rodopoulou et al. 2015; Zhang et al. 2018). For PM_2.5_, PM_10_, CO, SO_2_, and NO_2_, 24-h average concentrations were used; for O_3_, maximum daily 8-h average concentrations were used (Tian et al. 2018, 2019). During the same study period, we also obtained meteorological data, including daily mean temperature and relative humidity.

The study was approved by the ethics committee of the Beijing Tiantan Hospital. Since the data was analyzed at an aggregate level with no individual information involved, informed consent from the participants was waived for this study.

### Statistical analysis

In order to investigate the association between air pollution and hospital admissions for TIA, we used the generalized additive model with quasi-Poisson distribution in a time-series analysis (Hastie 1990; Nitta et al. 2010). We controlled long-time and seasonal trends by using a smoothing spline with 7 degrees of freedom (df) per year in the regression model. For the daily mean temperature and relative humidity, we used a smoothing spline with 3 df to control their effects on TIA admissions (Tian et al. 2018). The details of the smoothing spline have been described in previous literature (Hastie 1990). It is understood that day of the week (DOW) and public holidays may have an effect on hospital admissions; therefore, we included the DOW and public holidays as dummy variables in the model. Consequently, the regression model was constructed:

$$ \mathrm{In}\left[\mathrm{E}\left({Y}_t\right)\right]={\beta}_0+{\beta}_1\left( ai\mathrm{r}\  pollutants\right)+{\beta}_2 DOW+{\beta}_3 Holiday+{s}_1\left( time, df=7/ year\right)+{s}_2\left( temp, df=3\right)+{s}_3\left( hum, df=3\right) $$

where E(*Y*_*t*_) is the expected daily counts of hospital admission for TIA on day *t*, DOW is the day of the week, Holiday indicates a public holiday (0 = No, 1 = Yes), time is calendar time, temp is the daily mean temperature of the current day, hum is the relative humidity of the current day, *β* is the regression coefficient, and *s* indicates a cubic smoothing spline.

To investigate the lag effect associated with air pollutants, we used the following lag periods: single-day lag (the same day [lag 0], the previous day [lag 1], and the day before the previous day [lag 2]), multi-day lags (average concentration of the same day and previous day [lag 01], and average concentration of the same day and previous 2 days [day 02]). Additionally, we used a restricted cubic spline with 3 knots at the 5th, 50th, and 95th percentiles to explore the exposure-response relationship between air pollutants and hospital admissions for TIA.

We performed a subgroup analysis to evaluate whether the following subgroups were potential effect modifiers: gender (men or women), age (< 60 years or ≥ 60 years), temperature (cool days: < 15.7 °C, warm days: ≥ 15.7 °C; median temperature used as the cutoff,) and season (April to September, warm season; October to March, cool season). We used the *Z* test to compare differences in the association between subgroups (Altman and Bland 2003).

In the sensitivity analysis, we used the two pollutant models to estimate the association between the concentration of air pollutants and TIA admissions. Additionally, we used different df values for calendar time (5–10 per year), temperature (4–6), and relative moisture (4–6) to test the robustness of the results.

All results were presented as percentage changes and 95% confidence intervals (CIs) in daily hospital admissions for TIA per 10 μg/m^3^ increase in PM_2.5_, PM_10_, SO_2_, NO_2_, O_3_, and per 1 mg/m^3^ increase in CO. The analysis was conducted using the SAS version 9.4 (SAS Institute Inc, Cary, NC).

## Results

In total, there were 109,975 hospital admissions for TIA from 134 hospitals in Beijing, China, from 1 January 2014 to 31 December 2016. Table 1 summarizes the characteristics of daily hospital admissions for TIA, meteorological variables, and concentrations of air pollutants. On average, there were 100 admissions for TIA per day over the study period. Of the patients, 49.5% were men, and 71% were aged ≥ 60 years. The mean temperature was 13.8 °C, and the relative humidity was 53.1%. The daily 24-h mean (standard deviation [SD]) concentration was 79.1 (68.3) μg/m^3^ for PM_2.5_, 103.8 (74.9) μg/m^3^ for PM_10_, 1.2 (1.0) mg/m^3^ for CO, 50.6 (24.3) μg/m^3^ for NO_2_, 14.4 (17.3) μg/m^3^ for SO_2_, and 110.4 (72.4) μg/m^3^ for O_3_, respectively. The concentrations of PM_2.5_, PM_10_, CO, NO_2_, and SO_2_ were positively correlated with each other, with Spearman correlation coefficients between 0.52 and 0.87 (*P* < 0.001). Meanwhile, O_3_ was negatively correlated with each of the other pollutants (Table 2).

**Table 1:** Distribution of daily hospital admissions, meteorological factors, and air pollutants in Beijing, China, from 2014 to 2016

				Percentile				
Variable	Mean	Standard deviation	Minimum	P25th	P50th	P75th	Maximum	IQR
TIA admission	100.3	34.9	21	63	108	127	197	64
Meteorological factors								
Temperature, °C	13.9	11.0	− 14.3	3.0	15.7	24.1	32.6	21.1
Relative humidity, %	52.7	20.0	8.0	37.0	53.0	69.0	99.0	32.0
Air pollutants								
PM_2.5_, μg/m^3^	79.1	68.3	5.2	29.8	60	106.4	477.5	76.6
PM_10_, μg/m^3^	103.8	74.9	1.7	47	86.8	135.9	480.8	88.9
CO, mg/m^3^	1.2	1.0	0.2	0.6	1.0	1.5	8.1	0.9
NO_2_, μg/m^3^	50.6	24.3	8.1	33.5	44.5	61.4	153.5	27.9
SO_2_, μg/m^3^	14.4	17.3	1.8	3.6	7.9	17.4	133.1	13.8
O_3_, μg/m^3^	110.4	72.4	3.0	57.0	92.0	158.5	343.0	101.5

IQR indicates inter-quartile range; PM_2.5_, particulate matter with aerodynamic diameter < 2.5 μm; PM_10_, particulate matter with aerodynamic diameter < 10 μm; CO, carbon monoxide; NO_2_, nitrogen dioxide; SO_2_, sulfur dioxide; O_3_, ozone

**Table 2:** Spearman correlation coefficients among ambient air pollutants

	PM_2.5_	PM_10_	CO	SO_2_	NO_2_	O_3_
PM_2.5_	1.00	0.87^*^	0.83^*^	0.52^*^	0.79^*^	− 0.11^*^
PM_10_	-	1.00	0.73^*^	0.53^*^	0.78^*^	− 0.07†
CO	-	-	1.00	0.61^*^	0.82^*^	− 0.36^*^
SO_2_	-	-	-	1.00	0.63^*^	− 0.33^*^
NO_2_	-	-	-	-	1.00	− 0.34^*^
O_3_	-	-	-	-	-	1.00

*P < 0.001

†*P* < 0.05

PM_2.5_ indicates particulate matter with aerodynamic diameter < 2.5 μm; PM_10_, particulate matter with aerodynamic diameter < 10 μm; CO, carbon monoxide; NO_2_, nitrogen dioxide; SO_2_, sulfur dioxide; O_3_, ozone

**Fig. 1:**
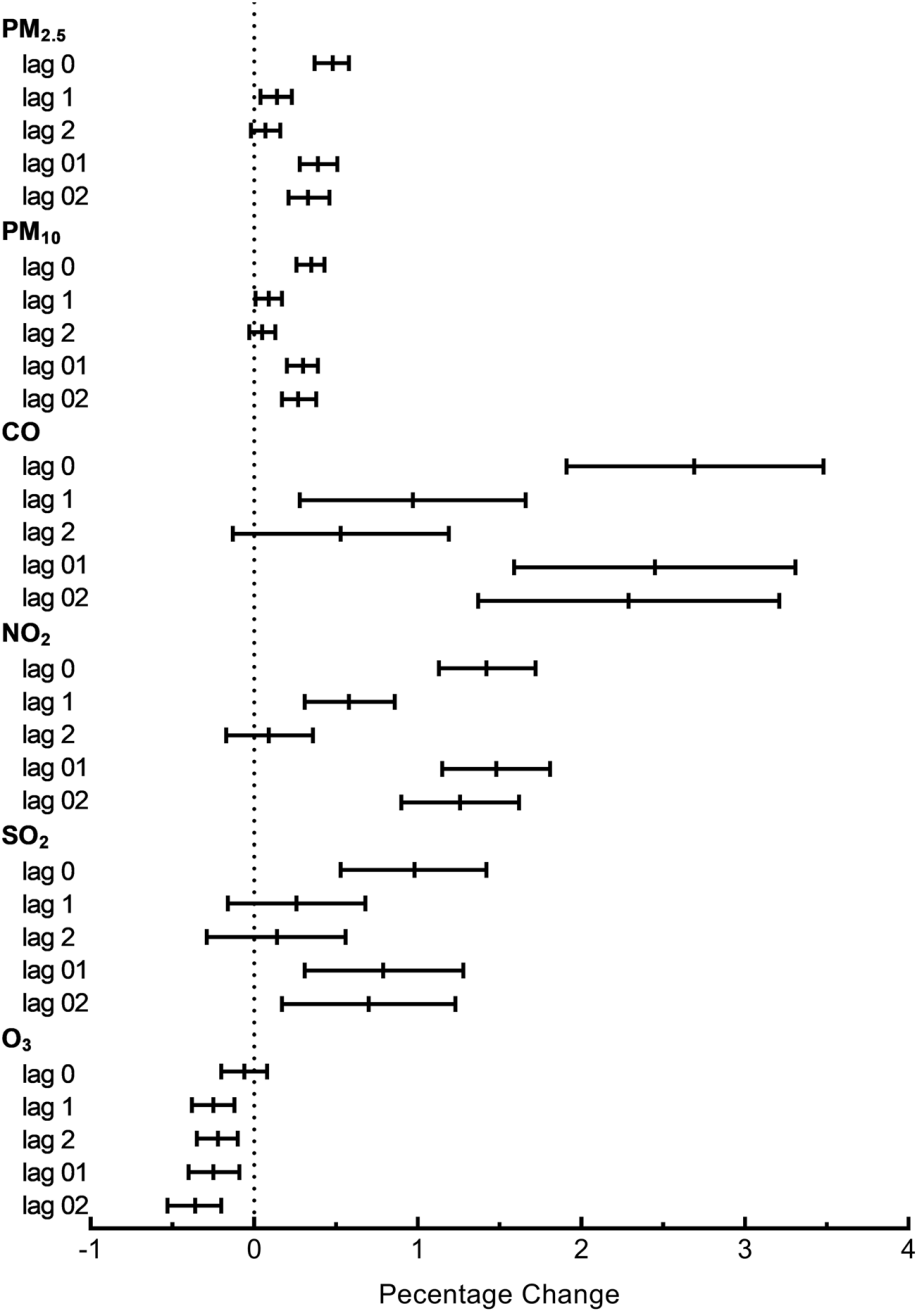
Percentage change and 95% confidence interval in daily hospital admissions for TIA per 10 μg/m^3^ increase in PM_2.5_, PM_10_, NO_2_, SO_2_, O_3_, and 1 mg/m^3^ increase in CO concentrations at different lag days

Figure 1 presents the percentage changes in hospital admissions for TIA on different lag days. With the exception of O_3_, the concentrations of PM_2.5_, PM_10_, CO, NO_2_, and SO_2_ on the same day (lag 0 days) showed the highest and significant associations with hospital admissions for TIA. The effects were attenuated at the other lag days. A 10 μg/m^3^ increase of PM_2.5_, PM_10_, NO_2_, SO_2_, and O_3_ in the same day and 1 mg/m^3^ increase in CO were associated with 0.48% (95% CI, 0.37–0.58%), 0.35% (95% CI, 0.26–0.43%), 1.42% (95% CI, 1.13–1.72%), 0.98% (95% CI, 0.53–1.42), − 0.06% (95% CI, − 0.2–0.08%), and 2.69% (95% CI 1.91–3.48%) increases in hospital admissions for TIA, respectively.

Table 3 shows the percentage change in hospital admissions for TIA per 10 μg/m^3^ increase in PM_2.5_, PM_10_, NO_2_, SO_2_, and O_3_ and 1 mg/m^3^ increase in CO concentrations on the same day, stratified by sex, age, temperature, and season. We observed that sex had a modifying effect on the association between PM_2.5_ and PM_10_ and hospital admissions for TIA; the association was stronger for men. For all pollutants, we did not find evidence of a modifying effect resulting from age. The associations between PM_2.5_, PM_10_, SO_2_, and O_3_ and admissions for TIA were more pronounced on warm days than cool days. However, we did not find significant effect modification by season for most pollutants, except for O_3_.

**Table 3:** The percentage change with 95% confidence interval in hospital admissions for TIA with 10 μg/m^3^ increases in PM_2.5_, PM_10_, NO_2_, SO_2_, O_3_, and 1 mg/m^3^ increase in CO on lag 0 days stratified by sex, age, temperature, and season

Pollutants	Subgroups	Percentage change	95% CI	*P*-value for effect modifications
PM_2.5_	Men	0.59	0.440.74	0.035
	Women	0.36	0.21–0.51	
	Age < 60	0.59	0.39–0.79	0.202
	Age ≥ 60	0.44	0.31–0.56	
	Cool days	0.39	0.25–0.52	0.015
	Warm days	0.68	0.49–0.87	
	Cool seasons	0.53	0.30–0.76	0.268
	Warm seasons	0.38	0.25–0.52	
PM_10_	Men	0.47	0.35–0.59	0.004
	Women	0.22	0.10–0.34	
	Age < 60	0.37	0.21–0.53	0.764
	Age ≥ 60	0.34	0.24–0.44	
	Cool days	0.28	0.17–0.39	0.019
	Warm days	0.5	0.35–0.66	
	Cool seasons	0.29	0.18–0.40	0.623
	Warm seasons	0.34	0.16–0.52	
CO	Men	2.91	1.80–4.03	0.613
	Women	2.50	1.40–3.61	
	Age < 60	3.04	1.59–4.51	0.597
	Age ≥ 60	2.57	1.64–3.51	
	Cool days	2.63	1.69–3.57	0.239
	Warm days	3.86	2.04–5.7	
	Cool seasons	2.60	1.65–3.56	0.700
	Warm seasons	3.02	1.13–4.94	
NO_2_	Men	1.50	1.08–1.92	0.613
	Women	1.35	0.93–1.77	
	Age < 60	1.51	0.96–2.06	0.714
	Age ≥ 60	1.39	1.04–1.74	
	Cool days	1.37	1–1.74	0.638
	Warm days	1.54	0.93–2.16	
	Cool seasons	1.32	0.94–1.70	0.862
	Warm seasons	1.39	0.68–2.10	
SO_2_	Men	1.36	0.73–1.99	0.097
	Women	0.61	− 0.02–1.24	
	Age < 60	0.88	0.06–1.70	0.769
	Age ≥ 60	1.03	0.50–1.55	
	Cool days	0.77	0.26–1.28	0.027
	Warm days	2.44	1.05–3.85	
	Cool seasons	0.78	0.27–1.29	0.084
	Warm seasons	2.13	0.69–3.60	
O_3_	Men	− 0.04	− 0.23–0.16	0.755
	Women	− 0.08	− 0.28–0.11	
	Age < 60	− 0.07	− 0.32–0.19	0.962
	Age ≥ 60	− 0.06	− 0.22–0.10	
	Cool days	− 0.9	− 1.26–− 0.54	< 0.001
	Warm days	0.23	0.07–0.4	
	Cool seasons	− 0.59	−0.92–0.26	< 0.001
	Warm seasons	0.13	− 0.03–0.29	

PM_2.5_ indicates particulate matter with aerodynamic diameter < 2.5 μm; PM_10_, particulate matter with aerodynamic diameter < 10 μm; CO, carbon monoxide; NO_2_, nitrogen dioxide; SO_2_, sulfur dioxide; O_3_, ozone

**Fig. 2:**
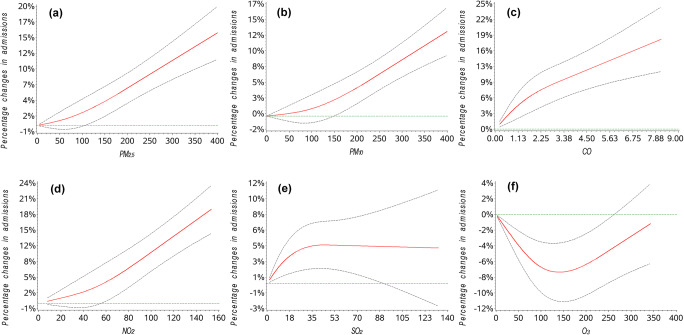
Adjusted exposure-response association curves between the concentrations of PM_2.5_, PM_10_, CO, SO_2_, NO_2_, and O_3_ and percentage changes in hospital admissions for TIA

Figure 2 displays the exposure-response curves for the associations between air pollutants and hospital admissions for TIA, adjusted for time trend, week effect, holiday, and meteorological variables. We observed almost linear trends for PM_2.5_, PM_10_, CO, and NO_2_. The curve increased steeply when the concentration of SO_2_ fell below 35 μg/m^3^ and then leveled off when the concentration rose above 35 μg/m^3^. The exposure-response association curve between O_3_ and hospital admissions for TIA was U-shaped.

In the two pollutant models, the associations were still significant for PM_2.5_ and NO_2_ after adjusting for other pollutants. In contrast, associations were diminished for PM_10_ and CO after adjusting for PM_2.5_ and NO_2_. The effect of SO_2_ became insignificant after adjusting for PM_2.5_, PM_10_, and CO. The relationships were insignificant for O_3_ after adjusting for PM_10_, CO, NO_2_, and SO_2_ (Table 4). When changing the df value for time trend (5–10 per year), the associations between air pollutants and hospital admissions for TIA were not changed.

**Table 4:** Percentage change with 95% confidence interval in transient ischemic attack admission with air pollutants in two pollutant models

Variable	PM_2.5_	PM_10_	CO	NO_2_	SO_2_	O_3_
Adjusted for PM_2.5_	-	0.05 (− 0.13–0.23)	0.49 (− 0.73–1.73)	0.99 (0.55–1.43) *	− 0.29 (− 0.82–0.23)	− 0.15 (− 0.29–− 0.01) *
Adjusted for PM_10_	0.42 (0.19–0.65) *	-	1.23 (0.11–2.36) *	1.21 (0.75–1.67) *	− 0.06 (− 0.57–0.46)	− 0.1 (− 0.24–0.04)
Adjusted for CO	0.42 (0.25–0.59) *	0.24 (0.12–0.37) ^*^	-	1.4 (0.97–1.83) *	− 0.08 (− 0.62–0.46)	− 0.02 (− 0.16–0.12)
Adjusted for NO_2_	0.21 (0.06–0.37) *	0.09 (− 0.05–0.22)	0.06 (− 1.06–1.2)	-	− 0.78 (− 1.33–− 0.24) *	− 0.01 (− 0.15–0.13)
Adjusted for SO_2_	0.51 (0.39–0.64) *	0.35 (0.25–0.45) *	2.77 (1.81–3.73) *	1.71 (1.35–2.08)*	-	− 0.04 (− 0.18–0.09)
Adjusted for O_3_	0.49 (0.38–0.60)*	0.35 (0.26–0.44)*	2.69 (1.90–3.48)*	1.42 (1.13–1.72)*	0.97 (0.52–1.41)*	-

*P < 0.05

PM_2.5_ indicates particulate matter with aerodynamic diameter < 2.5 μm; PM_10_, particulate matter with aerodynamic diameter < 10 μm; CO, carbon monoxide; NO_2_, nitrogen dioxide; SO_2_, sulfur dioxide; O_3_, ozone

## Discussion

To the best of our knowledge, this is the first study that systematically assessed the effects of short-term exposure to ambient air pollution on hospital admission for TIA in low- and middle-income countries. Our findings suggest that the concentrations of all analyzed air pollutants, except for O_3_, were positively associated with daily hospital admissions for TIA. Among different lag days, it was on the same day (lag 0) that the highest effects of PM_2.5_, PM_10_, CO, NO_2_, and SO_2_ on hospital admissions for TIA were observed. PM_2.5_ and PM_10_ had more pronounced effects in men, and the associations for PM_2.5_, PM_10_, SO_2_, and O_3_ on warm days were significantly stronger than on cool days.

In the past 20 years, the effects of air pollution on stroke have been explored in detail. However, there are few studies on TIA; this may due to the difficulty in accurately identifying TIA cases (Nadarajan et al. 2014; Sacco 2004). TIA has transience of symptoms and various clinical symptoms, and its diagnosis also relies heavily on an accurate interpretation of the patient’s clinical history (Schrock et al. 2012). The association between TIA and air pollution is not yet certain. A study from Canada found that exposure to SO_2_ during the summer months may increase the risk of TIA; however, no statistically significant associations between the other air pollutants and TIA were found (Villeneuve et al. 2006). From the Dijon Stroke Registry, a population-based study in France, a significant association was observed between O_3_ and TIA, but no associations were found for PM_10_, SO_2_, NO_2_, and CO (Henrotin et al. 2007). Another study was performed using the data from two UK cities; in Manchester, it was found that short-term change in concentration of NO on 3-lag day was positively associated with the occurrence of TIA and minor stroke. And, in Liverpool, PM_10_ on the same day, and NO on 3-lag day, was negatively associated with TIA and minor stroke (Bedada et al. 2012). However, the sample sizes of aforementioned studies were limited, and the data used were from developed countries with low-level air pollution. A recent study from 248 Chinese cities found that a 10 μg/m^3^ increase in PM_2.5_ was significantly associated with a 0.26% increase in hospital admissions for TIA (Gu et al. 2020). In our study, we found a much stronger effect: 0.48% changes on TIA admissions for a 10 μg/m^3^ increase in PM_2.5_. This difference may be due to the fact that the former study used data from a variety of locations, where the effects of PM_2.5_ may display different patterns (Chen et al. 2017; Liu et al. 2017). The former study represented the mean effect of 248 cities. Our study, which used the data from Beijing, on the other hand, represented an area with relatively severe air pollution, with a daily mean concentration of PM_2.5_ 79.1 μg/m^3^.

From the exposure-response curve, a nearly linear relationship can be observed between PM_2.5_, PM_10_, CO, and NO_2_ and hospital admissions for TIA. Few studies have examined the exposure-response relationship between air pollutants and TIA. In the previously mentioned study from 248 cities in China, the curve increased steeply at low concentrations of PM_2.5_ and reached a plateau at high concentrations (Gu et al. 2020). Differences in the shape of exposure-response curves may be a result of differences in geographic regions and estimation methods. In regard to the other pollutants, to our knowledge, this is the first study to explore exposure-response relationships between these pollutants and TIA. We should note that the number of observations for high levels of pollutants was small. Moreover, the restricted cubic spline is assumed to be linear beyond the extreme knots; we may observe an almost linear curve when assessing the whole range of pollutant levels. Therefore, we should use caution when deciphering the exposure-response curve. Future studies are needed to further estimate exposure-response associations, as well as explore the safety level if applicable.

In the subgroup analysis, we found that associations for PM_2.5_ and PM_10_ were more pronounced in women than in men. However, it was inconclusive whether or not gender is a modifier for the association between PM_2.5_ and PM_10_ and TIA. Regarding ischemic stroke, some studies have indicated that women are more vulnerable to air pollution (Franklin et al. 2007; Kan et al. 2008). The modifiable effect of sex for ischemic stroke was also unclear. More studies are warranted to further investigate this issue. Additionally, in our study, we found no evidence of age-specific differences in associations between air pollutants and admissions for TIA. This is consistent with the findings of the previous study (Gu et al. 2020) though the classification of age groups differs. Temperature may be another potential modifying factor. Previous studies have suggested that the association between particulate matters and stroke was higher on warm days than cold days (Huang et al. 2016; Tian et al. 2018). Similarly, we observed a stronger effect of PM_2.5_, PM_10_, SO_2_, and O_3_ on admissions for TIA on warm days than cold days. There were several potential explanations proposed for this increased association on warm days. First, the ambient temperature may influence the emission, transportation, dilution, chemical transformation, and deposition of air pollutants (Macdonald et al. 2005). Second, on warm days, people tend to spend more time outdoors (Tian et al. 2019), resulting in more exposure to ambient air pollution. However, when stratified by season, we observed no significant difference between associations in cool seasons and warm seasons. This was supported by the prior study (Gu et al. 2020).

In our study, we found a positive relationship between ambient air pollution and hospital admissions for TIA. We observed a 0.48% increase in hospital admissions for TIA per 10 μg/m^3^ increase in PM_2.5_. Although the magnitude of association appears to be relatively small, the public health burden derived from the risk could be highly significant. If the average concentration of PM_2.5_ was reduced from the current concentration of 79 μg/m^3^ to 10 μg/m^3^, which is the level proposed by the WHO air quality guidelines (Krzyzanowski and Cohen 2008), it was estimated that about 3.3 hospital admissions for TIA per day could be avoided in Beijing, China. The study strengthens the rationale for reducing concentrations of air pollutants in low- and middle-income countries.

Several limitations should be considered in our study. First, unlike ischemic stroke or hemorrhagic stroke, the diagnosis of TIA is difficult, because the symptoms and signs usually have resolved by the time of assessment (Nadarajan et al. 2014; Sheehan et al. 2009). Additionally, TIA mimics rate is higher (Nadarajan et al. 2014). The diagnosis for TIA may be less reliable. This is especially true when it comes to the administrative database; this may result in misclassification of the disease. In order to improve the accuracy of disease diagnosis, the Beijing Municipal Health Commission launches quality monitoring projects every year. However, misclassification is likely unrelated to air pollution and may reduce the precision of association or cause associations to be underestimated (Wellenius et al. 2005). Thus, our results should be explored with caution. Further research is needed to confirm the observed associations between air pollution and TIA admissions by examining these associations using a negative control cohort. Additionally, due to data limitations, we could not assess some potential modifiers, such as smoking, drinking, and individual disease history. We recommend that future research takes these potential modifiers into consideration in order to acquire more precise associations. Finally, exposure measurement errors may be present, since we used average exposure to air pollution as a proxy for true personal exposure. Measurement errors tend to lead the association to null (Zeger et al. 2000) and the conclusion to be more conservative.

## Conclusions

In conclusion, the study suggests that short-term exposure to air pollution is associated with hospital admission for TIA. Elevated levels of PM_2.5_, PM_10_, CO, NO_2_, and SO_2_ can result in an increase in hospital admissions for TIA. Gender and temperature may be the two potential effect modifiers on the association between air pollution and admission for TIA. Further research is needed to verify the expose-response between air pollution and admissions for TIA. This research contributes evidence on the association between air pollution and admissions for TIA in the low- and middle-income countries and may promote related public health policy development.

## Data Availability

The data on air pollution and meteorological factors can be obtained from the National Air Pollution Monitoring System (http://www.cnemc.cn) and the China Meteorological Data Sharing Service System (https://data.cma.cn). The data on admissions for TIA are available from the corresponding author upon reasonable request.
